# Metabolomic Dynamic Analysis of Hypoxia in MDA-MB-231 and the Comparison with Inferred Metabolites from Transcriptomics Data

**DOI:** 10.3390/cancers5020491

**Published:** 2013-05-03

**Authors:** I-Lin Tsai, Tien-Chueh Kuo, Tsung-Jung Ho, Yeu-Chern Harn, San-Yuan Wang, Wen-Mei Fu, Ching-Hua Kuo, Yufeng Jane Tseng

**Affiliations:** 1 Department of Pharmacy, National Taiwan University, No. 1, Jen-Ai Road, Section 1 Taipei 10051, Taiwan; E-Mail: f95423009@ntu.edu.tw; 2 The Metabolomics Group, National Taiwan University, Taipei 106, Taiwan; E-Mails: cot@cmdm.csie.ntu.edu.tw (T.-C.K.); pipisteve2@gmail.com (T.-J.H.); duke3d.harn@gmail.com (Y.-C.H.); sanyuan731@gmail.com (S.-Y.W.); 3 Center for Genomic Medicine, National Taiwan University, Taipei 10051, Taiwan; 4 Graduate Institute of Biomedical Electronic and Bioinformatics, National Taiwan University, Room 410 BL Building, No. 1, Roosevelt Road, Sec. 4, Taipei 106, Taiwan; 5 Department of Computer Science and Information Engineering, National Taiwan University, No. 1, Sec. 4, Roosevelt Rd., Taipei 10617, Taiwan; 6 Graduate Institute of Networking and Multimedia, National Taiwan University, No. 1, Sec. 4, Roosevelt Rd., Taipei 10617, Taiwan; 7 Department of Pharmacology, National Taiwan University, 11 F No. 1 Sec. 1, Ren-ai Rd., Taipei 10051, Taiwan; E-Mail: wenmei@ntu.edu.tw

**Keywords:** ^1^H-NMR spectroscopy, metabolic network, metabolomics, multivariate analysis, tumor hypoxia

## Abstract

Hypoxia affects the tumor microenvironment and is considered important to metastasis progression and therapy resistance. Thus far, the majority of global analyses of tumor hypoxia responses have been limited to just a single omics level. Combining multiple omics data can broaden our understanding of tumor hypoxia. Here, we investigate the temporal change of the metabolite composition with gene expression data from literature to provide a more comprehensive insight into the system level in response to hypoxia. Nuclear magnetic resonance spectroscopy was used to perform metabolomic profiling on the MDA-MB-231 breast cancer cell line under hypoxic conditions. Multivariate statistical analysis revealed that the metabolic difference between hypoxia and normoxia was similar over 24 h, but became distinct over 48 h. Time dependent microarray data from the same cell line in the literature displayed different gene expressions under hypoxic and normoxic conditions mostly at 12 h or earlier. The direct metabolomic profiles show a large overlap with theoretical metabolic profiles deduced from previous transcriptomic studies. Consistent pathways are glycolysis/gluconeogenesis, pyruvate, purine and arginine and proline metabolism. Ten metabolic pathways revealed by metabolomics were not covered by the downstream of the known transcriptomic profiles, suggesting new metabolic phenotypes. These results confirm previous transcriptomics understanding and expand the knowledge from existing models on correlation and co-regulation between transcriptomic and metabolomics profiles, which demonstrates the power of integrated omics analysis.

## 1. Introduction

Previous studies have revealed the impact of tumor hypoxia on the malignant properties and propagation features of cancer [1]. Significant correlations between overexpression of hypoxia-inducible factor-1α (HIF-1α) and patient mortality have been found in brain, cervical, oropharyngeal, ovarian, lung, colorectal, and endometrial tumors [2,3]. Erb *et al.* studied the different grades of malignancy in oligodendrogliomas [4], the results showed that the most discriminate metabolic markers are associated with tumor hypoxia. Chan *et al.* used patient biopsies to investigate specific biomarkers for colorectal cancer. They observed that the most characteristic markers to discriminate colorectal tumor from normal tissue are hypoxia-related metabolites [5].

Breast cancer is one of the leading causes of cancer death in women, and worldwide approximately one in nine women suffer from this malignant disease [6]. Many groups have used gene expression microarrays to study breast cancer tissue at the transcriptome level. It has been shown that the hypoxic microenvironment induces the expression of more than 100 genes which alter the tumor vitality, propagation, malignant progression, metastasis and resistance to chemotherapy or radiation treatment [7,8,9,10,11]. Similarly, protein arrays and proteomic profiling have been used to a more limited extent to study alterations at the proteome level. The most important molecules are hypoxia-inducible factors (HIFs), including oxygen labile α-subunit and stable β-subunit [6,12,13]. Under hypoxic condition, HIFα subunit (HIF1α, 2α, or 3α) becomes stabilized and dimerized with the HIF1β subunit to form the HIF heterodimeric complex, which binds to the so-called hypoxia responsive element (HRE) within target genes to activate gene expressions that change the nature of tumor cells [14].

Compared to genomics and proteomics, metabolomics is an emerging science. It is the omic science which quantifies the dynamic multiparametric metabolic response of living systems to patho-physiological stimuli or genetic modifications [15]. Metabolomics studies use high field nuclear magnetic resonance spectroscopy (NMR) or mass-based spectrometry techniques in conjunction with chemometric methods to construct representative metabolic profiles for different physiological states. The metabolite profile comprises hundreds to thousands of endogenous organic metabolites. Comparing the disturbances in these profiles with the basal metabolic state may reveal potential biomarkers for physiological stimuli [16]. There are few studies that have investigated the metabolite biotransformations in breast cancer or tumor hypoxia by NMR-based metabolomics [17,18,19]. Richardson *et al.* investigated the central carbon metabolism in breast cancer cell lines. The flux of carbon revealed the cellular transformation in different metabolic pathways [20]. Morse *et al.* characterized choline-related metabolites in breast cancer cell lines and *in vivo* animal models [21]. Weljie *et al.* studied the metabolic changes of breast cancer *in vitro* and *in vivo* [22]. Amino acids such as leucine, threonine, lysine, phenylalanine, and pyruvate, lactate were found to be influenced by hypoxia. Troy *et al.* compared the metabolic profiles of HIF-1β-deficient and wild type Hepa-1 cells under hypoxia. From the changes in the quantities of amino acid- and choline-related compounds, they found that HIF-1 is not the only mechanism that regulates glycolysis [23].

Systems biology helps researchers reveal the complex mechanisms behind biological systems. Researches integrating multiple omics data offer insight into complex biological effects by systems biology approaches. Zhu *et al.* developed a network reconstruction approach to construct probabilistic networks with multiple omics data including metabolomics and transcriptomics data of yeast. They employed a causal regulator detection algorithm to identify the causal regulators in the networks [24]. It also showed that systems biology is useful in cancer research. Jain *et al.* identified the key role of glycine in rapid cancer proliferation by integrating metabolomics data with preexisting gene expression data [25].

Breast cancer is currently the leading cause of womans’ death in many countries, and hypoxia is strongly associated with cancer progression and malignancy. Tumor hypoxia has been thoroughly investigated at the transcriptomic level for breast cancer. Currently, many studies focus on and discuss mechanisms of tumor hypoxia in breast cancer at the transcriptomic level. To better understand system responses to hypoxic perturbations in breast cancer, we designed a metabolic experiment to compare and integrate metabolomic and transcriptomic changes in a breast cancer cell under hypoxic conditions. We analyzed the metabolic changes that occurred as a function of time in MDA-MB-231 breast cancer cells undergoing hypoxia and normoxia by ^1^H-NMR. The metabolic differences were analyzed by principal component analysis (PCA) and were compared with the bioinformatics networks constructed in this study. To understand the predictive performance of identified metabolites, support vector machines (SVM) [26] are used to construct the prediction models. This study aims to integrate transcriptomic and metabolomic data to increase our understanding of tumor hypoxia in breast cancer.

## 2. Results and Discussion

### 2.1. NMR Metabolic Profiles

**Figure 1:**
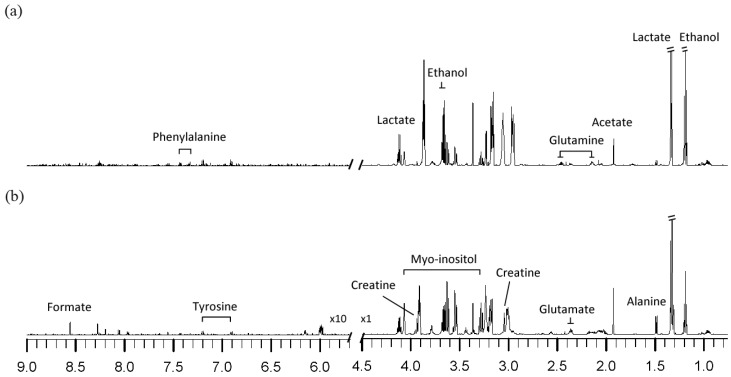
^1^H-NMR spectra of cell extracts obtained from MDA-MB-231 after 48h treatments under (**a**) hypoxia, (**b**) normoxia.

^1^H-NMR spectra of cell extracts obtained from the MDA-MB-231 breast cancer cell line under hypoxia and normoxia after 48 h are shown in Figure 1. The metabolites identified from the ^1^H-NMR spectrum included fatty acid (formate), carbohydrates (acetate, ethanol, lactate) and amino acids (alanine, phenylalanine, glutamate, glutamine, tyrosine), which are involved in the different metabolic pathways, such as energy metabolism (acetate, formate) and carbohydrate metabolism (ethanol, lactate).

Comparing the metabolite profiles of hypoxic to normoxic-treated cells, the metabolite levels of lactate, glutamine, and phenylalanine are increased, while those of myo-inositol, formate, tyrosine, creatine, glutamate, and alanine are decreased in hypoxic cells.

### 2.2. Influence of Hypoxia on Metabolic Profiles of MDA-MB-231 Cancer Cells

#### 2.2.1. Temporal Changes in the Metabolic Pattern Induced by Hypoxic Treatment in MDA-MB-231 Breast Cancer Cells

**Figure 2:**
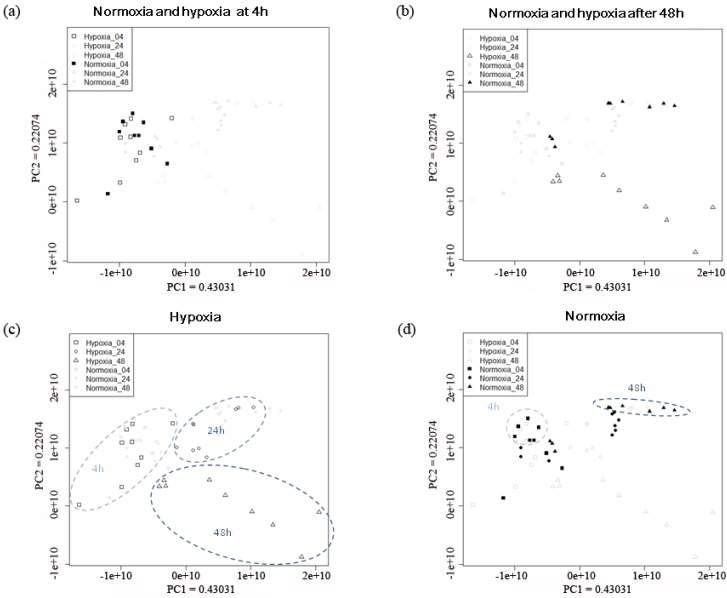
PCA scores plots of MDA-MB-231 breast cancer cells during extended periods of hypoxia and normoxia. (**a**) normoxia and hypoxia group with 4 h treatment was highlighted. (**b**) normoxia and hypoxia group after 48 h treatment was highlighted (**c**) hypoxia group at different time points was highlighted. (**d**) normoxia group at different time points was highlighted. The values of principal component 1 (PC1) and principal component 2 (PC2) on the axes indicated the proportion of variance explained by each of the principle component. Normoxia with 4 h treatment (■, n=9); 24 h treatment (●, n=9); 48 h treatment (▲, n=9). Hypoxia with 4 h treatment (□, n=9); 24 h treatment (○, n=9); after 48 h treatment (△, n=9).

The PCA results of the NMR spectra from MDA-MB-231 under hypoxic and normoxic treatment for 4 to 48 h are shown in Figure 2. The separation between hypoxia and normoxia was not obvious after 4 h treatment (Figure 2a). A clear separation with respect to hypoxia or normoxia treatment is clearly seen after 48 h treatment (Figure 2b). The trajectories of normoxic and hypoxic groups are in different directions. The trajectory of hypoxic group is shifted from the left to the lower right corner of the PCA plot (Figure 2c), whereas the trajectory of normoxic group is along the PC1 axis from 4 h to 48 h treatments (Figure 2d). Samples under 4 h normoxic condition separated from those under 48 h normoxic condition, and they were located on the opposite site of the PCA plot. Samples under 24 h normoxia located in the middle part of the plot and overlapped with both 4 h and 48 h normoxic samples. In contrast to the normoxic group, the samples exposed to 4 h, 24 h, and 48 h hypoxia showed distinct separation on the PCA plot. This observation indicates that there must be some specific metabolic differences between the two groups. Based on the result that 48 h treatment of hypoxia and normoxia showed distinct group separation (Figure 2b), we further compared the two 48 h-groups by PCA to investigate the metabolic differences.

**Figure 3:**
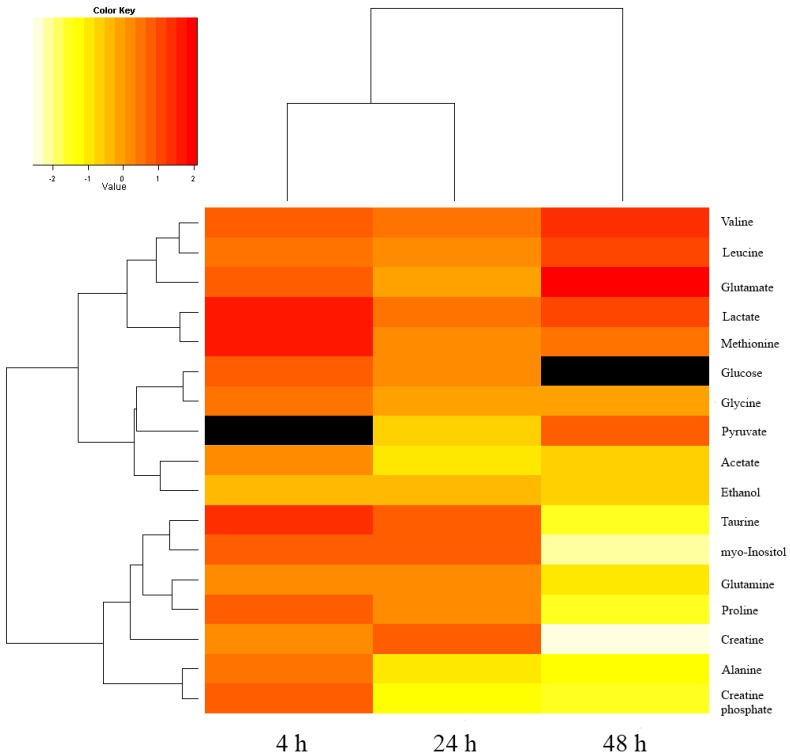
The quantified metabolites and fold changes in our study. The heatmap represents the log2-transformed fold changes for quantified metabolites with different treatments. The fold changes of pyruvate with 4 h treatment and glucose with 48 h treatment is not available, due to low concentrations (in black). n=3 for each group at each time point.

From the loading plot of 48 h hypoxic and normoxic samples, we identified 17 metabolites that contribute to the separation of the two groups (Figure S1 and Table S1). The relative concentrations of glutamine, valine, and leucine are about 2 to 4 times higher in the hypoxic samples. Methionine, lactate and pyruvate are only 1.5 to 2 times higher in the hypoxic group. The relative concentrations of myo-inositol, creatine, creatine phosphate, proline, and alanine are 2 to 6 times lower in the hypoxic group. Glutamate, glycine, acetate, and ethanol are 1.3 to 2 times lower in the hypoxic group. In addition, we traced back the changes of these 17 metabolites in the 24 h and 4 h treatment groups, and found that the fold changes of glutamine and valine increased with longer hypoxia treatment. In contrast, the fold changes of acetate, taurine, alanine, creatine phosphate, and myo-inositol decreased when the hypoxia treatment became longer. The main energy source-glucose-showed slightly higher concentration in hypoxic groups after 24 h and 4 h hypoxic treatment. However, the concentration of glucose between the two groups after 48 h treatment is too low to be detected. The fold changes of the 17 metabolites at different time points were calculated and the results are presented in Figure 3 and Table S2. Median and standard derivation of 17 metabolites were provided in the Table S3 to show the scatter of the metabolite levels.

To understand the predictive performance of 17 identified metabolites, a support vector machine (SVM) is used to construct the prediction models (Table 1). The result showed that the identified metabolites can predict hypoxia and normoxia well at 48 h treatments. The prediction of hypoxia at 48 h showed 83.33% (training data) and 100% (testing data) accuracy, specificity and sensitivity while the prediction performance, especially specificity, for 24 h treatment was much lower. These results suggested that the metabolic changes of hypoxia occurred between 24 and 48 h. Detailed explanation of SVM testing was provided in the supplementary information.

**Table 1:** The performance of predicting different time stages of hypoxia and normoxia in mixed samples.

	Training data (LOOCV ^b^)			
SVM Model ^a^	BAC ^c^	Accuracy ^d^	Specificity ^e^	Sensitivity ^f^
**4 h**	50.00%	33.33%	33.33%	33.33%
**24 h**	72.86%	66.67%	60%	71.43%
**48 h**	83.33%	83.33%	83.33%	83.33%
	**Testing data**			
**4 h**	66.67%	66.67%	66.67%	66.67%
**24 h**	66.67%	66.67%	33.33%	100%
**48 h**	100%	100%	100%	100%

^a^ Models were trained by linear SVM classifier, and each model was trained using two-third of all samples (n = 12). ^b^ Leave-one-out cross validation (LOOCV) was performed on training sets. ^c^ BAC denotes the average of specificity and sensitivity. ^d^ Accuracy = [(TN + TP)/(TP + FP + FN + TN)]. ^e^ Specificity = [TN/(TN + FP)]. ^f^ Sensitivity = [TP/(TP + FN)].

#### 2.2.2. Mechanism Discussion of Metabolic Changes

Tumor hypoxia affects tumor cell growth, apoptosis, differentiation, and metastasis by regulating several transcription factors such as hypoxia-inducible factor (HIF), nuclear factor (NF-κB), and p53 [27,28,29,30]. In addition, tumor hypoxia changes the cell metabolism by regulating the genes of metabolic enzymes such as lactate dehydrogenase (LDH), pyruvate kinase (PK), phosphoglycerate kinase (PGK), and glucose transporter (Glut). These changes increase the vitality and aggressiveness of tumor cell, and make tumor cell adapt to the severe hypoxic condition [31,32,33,34]. The exact values of the fold change were shown in Table S2. Both SVM testing results and the fold changes of metabolite concentration revealed the metabolic profiles between hypoxia and normoxia treatment were most distinct after 48 h treatment (Figure 3, Table S2). The differential metabolites at 48 h were further discussed to understand the effect of hypoxia on breast cancer cells, MDA-MB-231.

Lactate and pyruvate in the hypoxic group increased after 48 h treatment. This finding is coherent with the results of previous studies that hypoxia-inducible factor-1 (HIF-1) activates the genes encoded by glycolysis enzymes and glucose transporters under hypoxic condition [35,36,37,38,39]. The activation of glycolysis pathway increases the ATP production and finally increases the amount of pyruvate and lactate. The activation of LDH further converts pyruvate into lactate under hypoxic conditions [40]. However, the changes of pyruvate and lactate in MDA-MB-231 under hypoxic treatment are only 1.80- and 1.99-fold, which is less significant in comparison with other metabolites. This may be due to the nature of MDA-MB-231 that the expression of HIF-1α in metastatic MDA-MB-231 was found to be higher than other non-metastatic breast cancer lines (MCF-7) [41]. As HIF-1α was also induced in MDA-MB-231 after 48 h normoxic treatment, the concentrations of pyruvate and lactate were slightly increased. Therefore, the difference in fold changes of pyruvate and lactate between the two groups became small.

Glutamine and leucine are important amino acids which were found to be increased after hypoxia. Glutamine is the main source of nitrogen in tumor cells and is important for protein and DNA production. Breast cancer cells express the c-myc gene which regulates cell growth [42]. Myc regulates glutaminolysis and produces NADPH that is required for *de novo* nucleotide biosynthesis [43]. Besides, glutamine is also a major energy source other than glucose which is important in maintaining cell functions. Leucine is the precursor of adenosine triphosphate and is also essential to maintain cell proliferation. It was found that the uptake of radiolabeled glutamine and leucine in human neuroblastoma cells increased under hypoxic conditions [44]. The increase of glutamine and leucine in MDA-MB-231 after hypoxia treatment might be due to the increase of uptake in order to provide sufficient energy for cell proliferation. Acetate, alanine, proline, creatine phosphate, taurine, and myo-inositol decreased after 48 h hypoxic treatment. The biological roles and the possible mechanisms of the various metabolic changes are discussed in the following paragraphs. 

Acetate is the precursor of acetyl-CoA and fatty acids, and it plays an important role in cell metabolism. The incorporation of acetate into fatty acids has been investigated by radiolabeling methods. The results indicated that the incorporation of radiolabeled acetate into fatty acid increased under hypoxia in different cancer lines [45,46]. The increase of fatty acid synthesis is correlated with the activated expression of fatty acid synthetase (FAS) in tumor cells under hypoxic conditions [47,48]. In our study, the amount of acetate slightly decreased under hypoxia. This may be due to the increase of consumption of acetate to synthesize fatty acids.

The amount of alanine and proline decreased in MDA-MB-231 under hypoxia in our study. Alanine and proline are alternative sources of pyruvate [49]. Pyruvate is a key metabolite in metabolic network that could be converted to carbohydrate, fatty acid, and amino acid. Under anaerobic condition, glucose converts to pyruvate and is further metabolized to lactate to produce ATP and NAD^+^. NAD^+^ is then reused in highly activated glycolysis. The decrease of alanine and proline might be due to the conversion to pyruvate to increase the ATP production and NAD^+^ generation for further conversion to lactate [50].

Creatine phosphate was found to be decreased under hypoxic treatment. The decrease is proposed to compensate for the lower efficiency of ATP production. Creatine phosphate kinase converts creatine phosphate and ADP to creatine and ATP. This reaction generates ATP as another energy source [51]. The relative concentration of creatine phosphate was decreased significantly in MDA-MB-231 under hypoxic conditions to generate ATP for cancer proliferation.

The relative concentration of taurine in the hypoxic groups decreased as the duration of the hypoxic treatment became longer. Previous studies showed that taurine serves as an osmoregulator in brain, heart, and some tumor cells [52,53,54]. The hypo-osmotic insult induces the release of taurine from the cells. Schaffer *et al.* studied the relationship of chemical hypoxia and taurine release in neonatal cardiomyocytes. They concluded that the release of taurine in chemical hypoxic cells was caused by the increase of sodium in cells [55]. Under hypoxia, the microenvironment causes acidosis in tumor cell, and it was found that Na^+^/H^+^ exchangers were upregulated for extrusion of protons to adapt to this condition [56]. Based on these findings, we assume that the sodium content in MDA-MB-231 also increased under hypoxic treatment which resulted in the release of taurine from the tumor cells.

Myo-inositol is correlated with hormone signal transduction, detoxification, and osmoregulation under cell stress [57]. In this study the content of myo-inositol was decreased after 48 h hypoxic treatment underwent a −4.29-fold change. This was proposed to be caused by the consumption of myo-inositol as osmoregulator and detoxification substance when the severe hypoxic condition occurred.

### 2.3. Inference of Metabolic Pathway Network from Transcriptome and Metabolome

**Figure 4:**
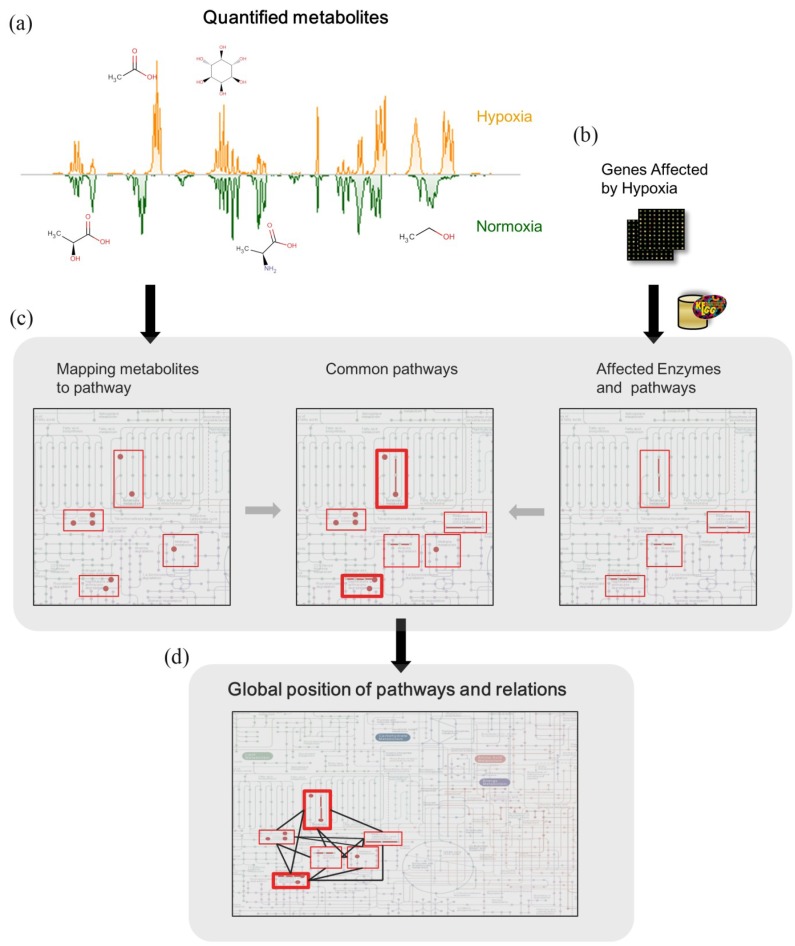
Metabolic network inference from transcriptomic data. (**a**) Quantified metabolites in our metabolomics study. (**b**) Genes affected by hypoxia in breast cancer cell were manually curated from literatures. (**c**) Enzymes corresponding to the genes and pathways affected by the corresponding enzymes were retrieved through KEGG KGML database. The pathways inferred from genomic data and quantified metabolites in our NMR spectra were listed and compared (Table 2). (**d**) To see the relations between pathways, pathways were further mapped to the KEGG global map, the complete result was shown in Figure 5.

In order to investigate the potential concordance from metabolomics study with pathways inferred from affected genes in other transcriptomics study, we proposed a metabolic pathway network method (Figure 4). The proposed method was as follows:

(1) Seventeen metabolites were quantified from our metabolomics study.

(2) We manually curated microarray data from the hypoxia study using the same cell line with this study [58]. Bando *et al.* investigated the up-regulated genes of MDA-MB-231after 1, 3, 6, 12 and 24 h of hypoxia. Out of 12,625 genes, 66 genes were considered up-regulated (down-regulated genes were not provided). The corresponding proteins of these 66 genes were retrieved through the KEGG Markup Language (KGML) database [59]. KGML files encompass the relationships between enzymes, compounds and the metabolic reactions in KEGG pathway maps. Enzymes and the corresponding metabolic reactions were extracted from KGML files by querying the Entrez gene ID of the 66 up-regulated genes, which were converted by the Database for Annotation, Visualization and Integrated Discovery (DAVID) program [60]. Twelve pathways were included in the metabolic reactions (Table 2).

**Table 2:** Pathways involved with metabolites from metabolome and transcriptome study. Metabolomics pathways inferred under hypoxia treatment were identified from our study. Transcriptomics pathways inferred under hypoxia treatment were obtained from the published study.

Pathway	Metabolome	Transcriptome
Glycolysis/Gluconeogenesis	V	V
Purine metabolism	V	V
Arginine and proline metabolism	V	V
Pyruvate metabolism	V	V
Pentose and glucuronate interconversions	V	
Ascorbate and aldarate metabolism	V	
Alanine, aspartate and glutamate metabolism	V	
Glycine, serine and threonine metabolism	V	
Cysteine and methionine metabolism	V	
Valine, leucine and isoleucine biosynthesis	V	
Taurine and hypotaurine metabolism	V	
D-Glutamine and D-glutamate metabolism	V	
Glutathione metabolism	V	
Nitrogen metabolism	V	
Fructose and mannose metabolism		V
Synthesis and degradation of ketone bodies		V
Arachidonic acid metabolism		V
Linoleic acid metabolism		V
Retinol metabolism		V
Metabolism of xenobiotics by cytochrome P450		V
Drug metabolism—cytochrome P450		V

(3) The metabolic reactions and corresponding pathways of 17 quantified metabolites were also collected from KGML files, where there were 14 pathways corresponding to our metabolomics study (Table 2).

**Figure 5:**
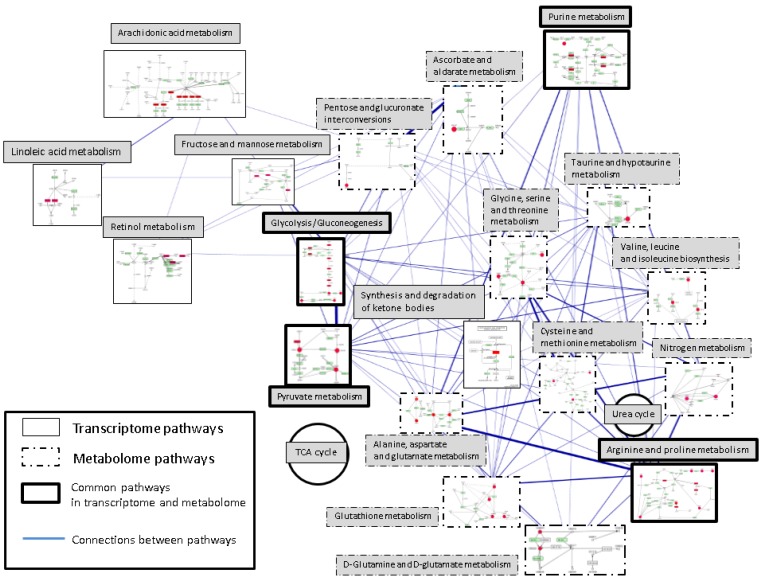
The pathway network of the metabolome and transcriptome. Metabolomics pathways inferred under hypoxia treatment were identified from our study. Transcriptomics pathways inferred under hypoxia treatment were obtained from the published study.Pathways on the right side of this figure including glycolysis/gluconeogenesis and pyruvate metabolism are the major changes in the metabolomic responses, and pathways on the left side including glycolysis/gluconeogenesis and pyruvate metabolism are the transcriptomic responses. Consistent pathways are “glycolysis/gluneogenesis”, “pyruvate metabolism”, “purine metabolism” and “arginine and proline metabolism”.Each rectangle represents one metabolism with the simplified pathway route. Pathways are placed according to the KEGG global metabolism map and connected according to the shared metabolites or enzymes. Mapped enzymes or metabolites are highlighted with red color. The widths of the edges represent the number of shared metabolites or enzymes between each two pathways pair. TCA and urea cycle are also placed on the map as two circles.

(4) To understand the differences between metabolomic and transcriptomic results, we compared the affected pathways of the metabolome and transcriptome. There were four common pathways: (glycolysis/gluconeogenesis, purine metabolism, arginine and proline metabolism, pyruvate metabolism). Also, to understand the relation between pathways, these pathways were laid out to specific position in the KEGG global metabolic map (Figure 5).

The proposed method could identify the consistent metabolic response from our direct identified metabolites and indirect metabolism pathways from expressed transcripts from literatures and past reports. Table 2 showed that the metabolism pathways from the quantified metabolites were highly related to the reported transcript literature results.

#### 2.3.1. Pathways in Common between Metabolome and Transcriptome

We mapped our identified metabolites and up-regulated genes from previous studies on KEGG reference pathways. Comparison of the mapped pathways of the metabolome and transcriptome displays consistent and incongruous pathways (see Table 2). Consistent pathways are “glycolysis/gluconeogenesis” (KEGG map id: 00010), “pyruvate metabolism” (KEGG map id: 00620), “purine metabolism” (KEGG map id: 00230) and “prginine and proline metabolism” (KEGG map id: 00330). Up-regulation of glycolysis/gluconeogenesis and pyruvate metabolism is well known in tumor hypoxia. Cells generate ATPs by the glycolysis pathway instead of entering TCA cycle and electron transfer chain. The mapped purine metabolism metabolites are glutamine and glycine. These two metabolites are located on the periphery of the purine pathway map, connecting to “glycine, serine and threonine metabolism” (KEGG map id: 00260) or participating very upstream of purine synthesis. The enzyme mapped on is the pyruvate kinase, muscle (PKM2, NCBI GeneID: 5315, E.C.: 2.7.1.40), which catalyzes transferring phosphorus-containing groups reactions. Therefore, direct correlation from this mapped pathway to tumor hypoxia is not obvious. Expression of PKM2 has been demonstrated as necessary to promote aerobic glycolysis with reduced oxidative phosphorylation and provides growth advantages for tumor cells [61]. It may be a pathway for expressing PKM2 to promote anaerobic glycolysis, which is also up-regulated under hypoxic conditions. In the arginine and proline metabolism, level of proline decreased at 48 h. As described in previous discussion, proline could be used as alternative energy source.

#### 2.3.2. Pathways Difference between Metabolic Profiling and Transcriptome

Pathways on the right side of Figure 5 including glycolysis/gluconeogenesis and pyruvate metabolism are the major changes in the metabolomic responses. The difference includes amino acid metabolisms (glutamine, glutamate, alanine, glutathione and other amino acids, see Table 2) and nitrogen metabolism. In contrast, pathways on the left side of Figure 5 including glycolysis/gluconeogenesis and pyruvate metabolism reflect the transcriptomic responses. These pathways contain fructose and mannose metabolism, lipid metabolisms (arachidonic acid metabolism and linoleic acid metabolism) and retinol metabolism. The connections indicated the shared metabolites and enzymes in a pair of two metabolic pathways. Wider edge stands for more metabolites and enzymes shared in two connected pathways. The mapped metabolomic pathways are much higher interconnected than the mapped transcriptomic pathways (metabolomic pathway: 75 connections/91 pathway pairs, transcriptomic pathways: eight connections/eight pathway pairs). The connection between metabolomic pathways (32 connections) and the consistency pathways is also higher than that between transcriptomic pathways and the consistency pathways (three connections).

Like the dynamic Pachinko model [62], transcriptome and proteome can reveal how the metabolome transforms. The system is probabilistic determined by thermodynamics and kinetics. The outcome of the entire system is dependent on the collisions of molecules and previous metabolic transformation. Knowing the pins (key enzymes or transporter) and holes (the exit of the system) on the Pachinko model may not be enough for understanding the entire system. However, the metabolome points out the end phenotypes against stimuli and provides valuable information. The metabolome is needed to realize the entire system incorporating other omics data.

## 3. Materials and Methods

### 3.1. Cell Culture and Hypoxia Treatment

MDA-MB-231 Breast Cancer Cells were seeded onto a 10 cm dish at a density of 8 × 10^5^ cells per dish. The cells were maintained in RPMI 1641 medium (Gibco^TM^-Invitrogen, Grand Island, NY, USA) with the addition of sodium bicarbonate, 10% fetal bovine serum, 1% penicillin and 1% streptomycin at 37 °C under humidified atmosphere containing 5% CO_2_ at 37 °C. After 2 days, the growth medium was replaced with fresh growth medium and the cells were placed in hypoxia chamber or normal incubator. For hypoxic treatment, cells were grown at 37 °C in a Hypoxia chamber (Anaerobic System PROOX model 110; BioSpherix, Redfield, NY, USA) at 0.5% O_2_ with a gas mixture consisting of 95% N_2_/5% CO_2_. 

### 3.2. Extraction of Intra-Cellular Metabolite

Cells were harvested at 4 h, 24 h and 48 h after hypoxia or normoxia treatment. The culture medium was quickly removed and the cells were washed twice with 2 mL ice-cold PBS. After washing with PBS solution, the remaining cells were placed on ice and suspended in 600 mL ice-cold D_2_O for 30 min. D_2_O extract was transferred to Eppendorfs and centrifuged at 12,000 g at 4 °C for 5 min. The supernatant was collected and stored at −80 °C until use. 

### 3.3. Sample Preparation for ^1^H-NMR Spectroscopy

Cell extract was thawed at room temperature. Two hundred and ninety-seven μL of cell extract was mixed with 3 μL of 5 mM sodium 3-trimethylsilyl-(2,2,3,3-^2^H_4_)-1-propionate (TMSP) in D_2_O (final concentration 0.05 mM). The D_2_O provided a NMR lock signal for the NMR spectrometer. One hundred micro-liter of the sample was then transferred into a 2 mm NMR tube. 

### 3.4. Metabolome Analysis

#### 3.4.1. NMR Analysis

Conventional ^1^H-NMR spectra of the cell extraction samples were obtained using a Bruker Avance 600 spectrometer (Bruker Biospin, Germany) operated at 600.04 MH_Z_ at 25 °C. The one-dimensional ^1^H-NMR spectra were acquired using a standard NOESYPR1D pulse sequence (recycle delay-90°-t_1_-90°-tm-90°-acquisition; XWIN-NMR3.5) with a recycle delay time of 2 s, and a mixing time of 150 ms. The 90° pulse length was adjusted to ~4 μs at 0.17 dB and t_1_ was set to 4 μs, which provided an acquisition time of 2.72 s. For each sample, 128 free induction decays (FID) were collected using 32 k data points within 10 ppm, and the total data collecting time was 11 min. FIDs were then multiplied by an exponential weighting function corresponding to a line broadening of 0.3 Hz, and the data were zero-filled to 64 k data points.

#### 3.4.2. Metabolite Identification

All acquired FIDs were Fourier transformed, phase corrected and aligned to the chemical shift of the alpha-glucose anomeric doublet at 5.223 ppm using ACD/Labs v.10.0 1D NMR Manager (Advanced Chemistry Development, Inc., Toronto, ON, Canada). The FIDs were further imported into R v. 2.8.1 for water deletion, scaling, baseline correction and normalization [63]. The region of the peak containing H_2_O was removed within 4.5 to 5 ppm. Spectral intensities were scaled to the ratio of TMSP intensity at unit resolution in each NMR spectra. Then, an in-house baseline correction process and robust mean normalization were applied to each spectrum. The spectra region within 0.2 to 4.4 ppm was binned into 420 bins with a binning size of 0.01 ppm. Identification of metabolites was performed using the Profiler module of the Chenomx NMR Suite v.6.1 (Chenomx Inc., Edmonton, AB, Canada). The concentration of each metabolite was determined by Chenomx and was further normalized by the sum total concentration of each tested sample. Two-dimensional (2D) homonuclear j-resolved and total correlation spectroscopy (TOCSY) and heteronuclear multiple bond correlation (HMBC) NMR experiments were also used for peak assignment to specific metabolites.

### 3.5. Data Analysis

#### 3.5.1. Statistical Analysis

Principal component analysis (PCA) was performed on these preprocessed data with mean centered using the stats package in R. PCA score plot visualizes the clustering of hypoxic group *versus* normoxic group with different treatments. The significant levels of peak regions in spectrum were assessed by each Principle Component (PC) loading, e.g., the Euclidean distance of PC1 and PC2 loadings. The potentially significant peak regions were marked out by setting a threshold of significant level. 

To ensure the potentially significant peak regions were statistically significant, univariate analysis was performed on the integration of potential significant regions by paired-t test with α = 0.05 in R. 

The fold changes of the identified compounds between tumor cells under hypoxic to normoxic condition were further computed from the concentration retrieved above. For each samples in either the hypoxia or normoxia group, the median of the concentrations was selected as a representative of the group for the fold change analysis. To calculate fold changes, we apply the following procedure: (a) Let the fold changes equals to the ratio of the concentration in hypoxia to that in normoxia; (b) Set the fold changes in log2 scale. The positive fold changes mean the higher concentration in hypoxia, and the negative fold changes mean the higher concentration in normoxia.

#### 3.5.2. Classification Evaluation

To assess the predictive performance of identified metabolites, the concentration of identified metabolites were taken as features for each sample and predictive models were created using support vector machine (SVM). SVM maps the data from original feature spaces to high dimensions and searches for a hyper-plane to separate the hypoxia from the normoxia. We used the C interface to LIBSVM [64] to perform the training and test approach. 

There were total 54 samples, nine samples each from hypoxia and normoxia treatment for 4 h, 24 h and 48 h (Table 3). For the classification of hypoxia at 4 h, 24 h and 48 h, samples with hypoxic treatment were labeled as positive items for this classification model (n = 9 for each time point) and samples with normoxic treatment were labeled negative (n = 9 for each time point). Six hypoxic samples and six normoxic samples were randomly chosen as the training set for each hypoxic classification model at 4 h, 24 h and 48 h.

**Table 3:** Number of samples in each group.

	4 h	24 h	48 h
**Hypoxia**	9	9	9
**Normoxia**	9	9	9

Leave-one-out cross validation (LOOCV) was performed on the training set to tune the kernel parameters. Cross validation was conducted with respect to the average of specificity and sensitivity (BAC), and the predictive performance was reported as specificity [TN/(TN + FP)], sensitivity [TP/(TP + FN)] and accuracy [TN + TP/(TP + FP + FN + TN)]. 

#### 3.5.3. Construction of Pathway Network from Transcriptome and Metabolome

The transcriptomic data of MDA-MB-231 were extracted and curated from up-regulated genes under hypoxic condition [58]. The affected metabolic pathways of the selected transcriptomic data and the identified metabolites in this study were extracted via the Kyoto Encyclopedia of Genes and Genomes (KEGG) Pathway [65] (Release 53.0). Metabolic pathways were compared and organized from the affected pathways of the transcriptome and metabolome as Table 2. All affected pathways of the transcriptome and metabolome were chosen to construct metabolic pathway network. Each pathway was one single node. The shared metabolites and enzymes in one pathway-pathway pair were used as the edge. Wider edges represented the larger number of shared metabolites and enzymes in two pathways. The pathway network was visualized by Cytoscape 2.6.3 [66] and the relative position of each pathways was assigned according to KEGG global map (KEGG map id: 01100).

## 4. Conclusions

Our ^1^H-NMR spectroscopy experiments show the metabolic differences between hypoxia- and normoxia-treated MDA-MB-231. Through the PCA analysis, not only the discrimination of different condition groups is clear, but also the regions of NMR spectra which make the two groups different were discovered and verified by statistical analysis. Seventeen metabolites were quantified, which could be seen as potential hypoxic biomarkers to determine tumor hypoxic conditions.

Our time-dependent metabolic profile analysis showed that the discrimination between hypoxia and normoxia was ambiguous over 24 h but became clear over 48 h. This result suggests that the time point shown the change of metabolism might be between 24 and 48 h. In a previous transcriptomic study, the major changes were observed before 12 h [58]. As metabolomic changes became prominent after transcriptomic changes, these changes can be aligned with physiological consequences. 

Not only we can discover the metabolites associated with hypoxia and time-dependent biomarkers but also with the approach of a combined transcriptomics and metabolomics model, we constructed the metabolic pathway network with combining the metabolome and transcriptome for comparison of the mapped pathways of the two omics. The results indicated that there was some concordance in metabolism between the transcriptome and metabolome, but using a single platform would lead to only partial understanding of the hypoxia results. This integrated omics analysis could improve our understanding on the mechanisms of adaptation to hypoxia in breast cancer.

## Supplementary Files

Supplementary File 1 Supplementary Information (PDF, 117 KB)
