# Supplementary material for: Metabolomic Dynamic Analysis of Hypoxia in MDA-MB-231 and the Comparison with Inferred Metabolites from Transcriptomics Data
Source: Cancers (Basel). 2013 May 3;5(2):491–510. doi: 10.3390/cancers5020491 (PMC3730319; doi:10.3390/cancers5020491)
Supplement: Supplementary File 1 — Supplementary Information (PDF, 117 KB) [file cancers-05-00491-s001.pdf]

Supplementary Information

Identification of 17 Metabolites Contributed to the Separation of the Hypoxic and Normoxic Groups

Each NMR spectrum of samples were segmented into 420 chemical shift bins of 0.01 ppm from 0.2 to 4.4. Chemical shift bins were used as variables in principle component analysis (PCA). To search distinct variables contributing separation of hypoxic and normoxic group, samples with 48 hr treatment are chosen to perform PCA. The variables with similar behavior were aggregated in the loading plot of PCA. In contrast, the variables with distinct behavior were out of aggregation (Figure S1). Therefore, Euclidean distances from each variable to the central of all variables were used to determine the contribution to separation of hypoxic and normoxic group. Variances with more than 0.5 standard deviation distance were organized as range of chemical shift with the most distinct expression in hypoxic and normoxic group. For example, chemical shifts 1.17~1.21 consisted of 5 bins (1.17, 1.18, 1.19, 1.20 and 1.21). The potentially peak regions were marked out by setting a threshold of significant level.

Figure S1. The loading plot of principle component analysis in 48 h.

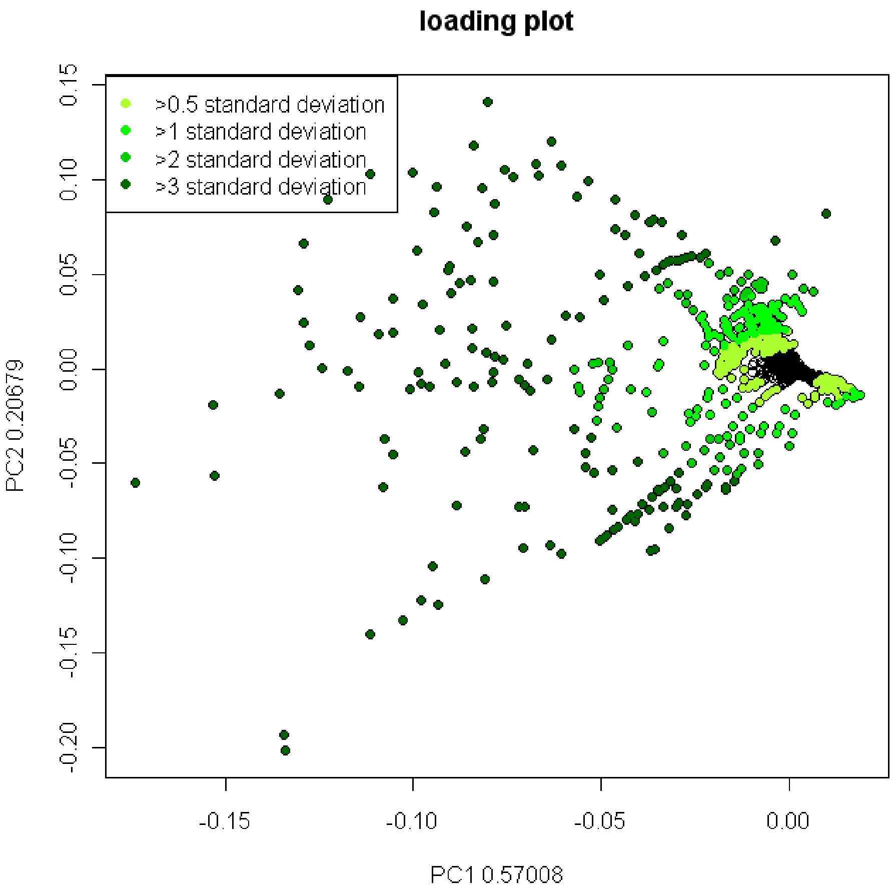

To ensure the statistical significance of each peak regions, univariate statistics analysis was performed on integration of potential peak regions by paired t-test with α = 0.05 (Table S1). Afterward, 17 metabolites are identified in the potential peak regions.

Table S1. The potential peak regions contributed to separation of hypoxic and normoxic group in 48 h treatment.

Chemical shift (PPM)	Identified metabolites	*P*-value of paired t-test
**0.97~0.97**	Leucine, Valine	0.009
**1.17~1.21**	Ethanol	0.012
**1.31~1.35**	Lactate	0.002
**1.47~1.49**	Alanine	1.154e-4
**1.93~1.93**	Acetate	0.001
**2.13~2.15**	Methionine	0.001
**2.35~2.37**	Pyruvate, Proline	0.003
**2.45~2.47**	Glutamine	7.042e-5
**2.91~3.29**	Creatine, Creatine phosphate	4.273e-4
**3.43~3.43**	Taurine, Glucose,<br>Proline, myo-insitol	5.553e-4
**3.53~3.55**	Glucose, Glycine, myo-insitol	1.551e-8
**3.61~3.65**	Ethanol, myo-insitol	7.512e-5
**3.77~3.93**	Glucose, myo-insitol	2.135e-4

Metabolites may be identified in multiple regions due to multiplet signals.

Table S2. Fold change of identified metabolites. Fold changes are ratio of metabolite expression in hypoxic group to normoxic group.

	**4 h**	**24 h**	**48 h**
**Acetate**	1.24	0.54	0.69
**Alanine**	1.47	0.56	0.39
**Creatine**	1.11	1.89	0.17
**Creatine phosphate**	1.57	0.45	0.34
**Ethanol**	0.72	0.76	0.64
**Glucose**	1.68	1.26	NA *
**Glutamate**	1.69	0.92	0.76
**Glutamine**	1.19	1.25	4.22
**Glycine**	1.45	1.03	0.51
**Lactate**	3.08	1.42	1.99
**Leucine**	1.45	1.17	2.17
**Methionine**	3.11	1.22	1.51
**Proline**	1.63	1.2	0.37
**Pyruvate**	NA *	0.62	1.8
**Taurine**	2.37	1.76	0.34
**Valine**	1.74	1.39	2.65
**myo-Inositol**	1.79	1.71	0.23

* The expression of pyruvate in 4 h and glucose in 48 h are not available due to low concentration. Fold change: median of expression in hypoxia group/median of expression in normoxia group.

Table S3. Median and standard derivation of identified metabolites at three time points.

	4 h Hypoxia	4 h Normoxia	24 h Hypoxia	24 h Normoxia	48 h Hypoxia	48 h Normoxia
**Acetate**	0.63 ±0.43	0.51 ±0.44	1.69 ±1.02	3.12 ±2.30	5.71 ±1.66	8.30 ±1.67
**Alanine**	0.42 ±0.25	0.29 ±0.14	0.51 ±0.29	0.9 ±0.83	1.06 ±0.42	2.70 ±0.81
**Creatine**	0.35 ±0.33	0.31 ±0.27	1.54 ±0.65	0.81 ±0.78	0.36 ±0.17	2.15 ±0.69
**Creatine phosphate**	0.53 ±0.19	0.34 ±0.14	0.25 ±0.27	0.55 ±0.21	0.25 ±0.08	0.75 ±1.40
**Ethanol**	8.81 ±4.97	12.27 ±15.59	14.35 ±4.65	18.79 ±13.14	22.1 ±8.72	34.39 ±18.58
**Glucose**	10.19 ±8.4	6.05 ±4.37	0.38 ±0.50	0.30 ±1.14	NA *	NA *
**Glutamate**	1.81 ±1.36	1.07 ±1.07	1.18 ±0.26	1.27 ±0.35	1.43 ±0.47	1.87 ±0.35
**Glutamine**	1.48 ±1.13	1.24 ±0.69	0.90 ±0.50	0.72 ±0.30	2.30 ±0.99	0.54 ±0.16
**Glycine**	0.68 ±0.38	0.47 ±0.26	0.90 ±0.36	0.88 ±0.32	0.64 ±0.15	1.25 ±0.14
**Lactate**	6.38 ±9.83	2.07 ±1.73	9.23 ±6.2	6.51 ±7.03	37.55 ±15.55	18.89 ±7.07
**Leucine**	0.61 ±0.3	0.42 ±0.14	0.36 ±0.11	0.31 ±0.08	1.31 ±0.52	0.60 ±0.19
**Methionine**	0.56 ±0.29	0.18 ±0.07	0.22 ±0.05	0.18 ±0.07	0.38 ±0.13	0.25 ±0.09
**Proline**	0.53 ±0.48	0.33 ±0.33	0.67 ±0.16	0.56 ±0.58	0.62 ±0.44	1.7 ±0.96
**Pyruvate**	NA *	NA *	0.15 ±0.41	0.25 ±0.33	0.39 ±0.25	0.22 ±0.19
**Taurine**	1.01 ±1.67	0.43 ±0.20	1.66 ±0.63	0.95 ±0.44	0.74 ±1.37	2.14 ±0.71
**Valine**	0.35 ±0.15	0.20 ±0.10	0.21 ±0.06	0.15 ±0.03	1.22 ±0.44	0.46 ±0.21
**myo-Inositol**	2.62 ±3.24	1.46 ±3.33	6.99 ±4.93	4.08 ±6.07	4.09 ±1.67	17.54 ±5.22

* The expression of pyruvate in 4 h and glucose in 48 h are not available due to low concentration.

Classification of Hypoxia *versus* Normoxia with Identified Metabolites

Total of 17 metabolites were considered significant between hypoxia and normoxia condition in our metabolomics study. The metabolic differences were analyzed by principal component analysis (PCA) and were compared with the bioinformatics network constructed in this study. To understand the predictive performance of identified metabolites, support vector machine (SVM) is used to construct the prediction models. For each predictive model, leave-one-out cross validation (LOOCV) was applied to the training set and the generalization was confirmed from the testing set.

The classification model performance of the 17 metabolites in both training and testing data was reported using statistical measures—specificity, sensitivity, and accuracy (Table 1). Sensitivity is the rate of tumor hypoxia which is correctly identified as hypoxia. Specificity is the rate of tumor normoxia which is correctly identified as normoxia.

Prediction of Hypoxia and Normoxia in Different Time Stages by SVM

There were 2 treatments (hypoxia and normoxia) and 3 different durations of treatments (4 h, 24 h and 48 h). Models were constructed from 3 time stages by SVM. To measure the performance of predicting hypoxia and normoxia in different time stages, all samples were relabeled to three classes: hypoxia against normoxia group at 4 h, 24 h and 48 h. For each class, two-thirds of all samples were randomly chosen for training data, and the remaining samples were used as testing data. The testing data were used to test whether the classification models from identified metabolites were results of overfitting. If the model overfit the data, the predictive performance on testing data would be worse than the LOOCV (Leave-one-out cross validation) on training data.

The linear SVM model at 4 h reported poor prediction (33% for sensitivity, specificity and accuracy). The prediction performance at 4 h suggested phenotypes between hypoxia and normoxia does not have

distinct differences. The prediction performance of model at 24 h was good for sensitivity reached 71.43% from 33.33% at 4 h. Although, the specificity is still low (60%). It suggested separation of samples with 24hr hypoxic/normoxic treatment are hard. The prediction performance at 48 h displayed model of 48 h can separate hypoxic and normoxic samples well for sensitivity, specificity, accuracy and balanced accuracy (BAC) are more than 80% (Table 1). The prediction performance at 24 h and 48 h displayed classification models are able to separate samples with hypoxia and normoxia treatment well.

Nevertheless, the specificity was low on both training and testing data for the hypoxia/normoxia prediction at 24 h treatment (60% specificity for training data and 33.33% for testing data). That is, samples at 24 h normoxic treatment was hard to be separated from those at 24 h hypoxic treatment. This may be contributed from the inconsistency of metabolic changes of hypoxia, *i.e.*, metabolism of part of the hypoxia samples had strongly changed while other biological mechanisms did not change obviously. The result was concordant to the PCA analysis, which suggested that the metabolism of hypoxia may change between 24 and 48 h.

The Consistent Metabolic Response in the Metabolome and Transcriptome

Through mapping our quantified metabolites and up-regulated genes from literature onto KEGG Pathway, the influenced network of the metabolic pathways were constructed. Glycolysis/Gluconeogenesis (KEGG map id: 00010), Pyruvate metabolism (KEGG map id: 00620), Purine metabolism (KEGG map id: 00230) and Arginine and proline metabolism (KEGG map id: 00330), four metabolisms are consistent in metabolomic and transcriptomic responses. It displayed that energy metabolism is the mainly regulated response under hypoxic condition from the aspects of transcriptome and metabolome levels. Purine metabolism is needed to generate nucleotides or ATPs for tumor cells. Altered levels of glutamine, glutamate, proline, creatine and creatine phosphate were detected in Arginine and proline metabolism. Nitric oxide synthase (NOS1) and propyl 4-hydroxylase (P4HA3) were up-regulated in Arginine and proline metabolism.

Major Difference between Metabolome and Transcriptome

Mapped metabolomic pathways include various amino acid metabolisms, such as glutamine, glutamate, alanine, asparatate and glutathionine metabolisms. The levels of amino acids increase at 4 h may response the hypoxia as a resistance mechanism to this acute stress. Abnormal or unsuitable proteins for hypoxia could be degraded as amino acids and re-synthesize proteins against hypoxia. At 24 h or 48 h, levels of amino acids become stable and some of amino acids are needed and used for different purposes as already stated in our previous discussion.

One of the three mapped enzymes on mapped transcriptomic pathways is Cytochrome P450, family 2, subfamily C (CYP2C19, NCBI GeneID: 1557, E.C.: 1.14.14.1). The up-regulation of CYP2C19 influenced “Arachidonic acid metabolism” (KEGG map id: 00590), “Linoleic acid metabolism” (KEGG map id: 00591) and “Retinol metabolism” (KEGG map id: 00830). Liu *et al*. demonstrated that prostaglandin E2 production from arachidonic acid is involved in hypoxia-induced vascular endothelial growth factor (VEGF) expression and regulated angiogenesis in prostate cancer. It suggests that there may be a similar mechanism of angiogenesis regulation in breast cancer. The other two mapped enzymes are hexokinase 2 (HK2, NCBI GeneID: 3099, E.C.: 2.7.1.1) and phosphofructokinase, muscle (PFKM,

NCBI GeneID: 5213, E.C.: 2.7.1.11) on “Fructose and mannose metabolism” (KEGG map id: 00051). Tumor cells may use fructose or mannose as the alternative glucose sources with up-regulated corresponded enzymes for reacting to increased glucose consumption (Warburg effect). From the difference of mapped metabolomic and transcriptomic pathways, the responses of metabolome are not dependent on the responses of transcriptome.
